# How important is the angle of tilt in the WHO cone bioassay?

**DOI:** 10.1186/s12936-016-1303-9

**Published:** 2016-04-27

**Authors:** Henry F. Owusu, Pie Müller

**Affiliations:** Epidemiology and Public Health Department, Swiss Tropical and Public Health Institute, Socinstrasse 57, 4051 Basel, Switzerland; University of Basel, Petersplatz 1, 2003 Basel, Switzerland

**Keywords:** Mosquitoes, Long-lasting insecticidal net, Insecticide resistance

## Abstract

**Background:**

The World Health Organization (WHO) cone bioassay plays an integral role in the evaluation of the efficacy of long-lasting insecticidal nets as well as insecticides used in indoor residual spraying. The test is used on a variety of treated substrates, such as pieces of bed nets, mud, cement and wood. The cone setup assumes a wide variety of angles under different settings in which it is applied. However, the guidelines provided for the performance of the assay do not specify the angle at which the test must be performed.

**Methods:**

Laboratory colonies of *Anopheles gambiae* Kisumu-1 and *Anopheles stephensi* STI were tested in the WHO cone bioassay at four different angles (0°, 45°, 60° and 90°) following the WHO guidelines against net pieces of Olyset Plus and Netprotect. The tests were repeated after 20 washes of the nets. Individual mosquitoes were also exposed at 0° and 60° and the amount of time each spent in contact with the net was recorded.

**Results:**

Mosquitoes spent more time on the net at 60° as compared to 0° (coefficient = 45.8, 95 % CI 34.6–55.6, p < 0.001) and were more likely to die when the test was done at 45° (OR 3.3, 95 % CI 1.7–6.3, p = 0.001), 60° (OR 3.1, 95 % CI 1.7–5.9, p < 0.001) and 90° (OR 6.0, 95 % CI 1.9–18.5, p = 0.002) as compared to 0°.

**Conclusion:**

The angle at which the test is performed significantly affects the amount of time mosquitoes spend resting on the nets, and subsequently mortality. Angle must thus be considered as an important component in the performance of the assay and duly incorporated into the guidelines.

**Electronic supplementary material:**

The online version of this article (doi:10.1186/s12936-016-1303-9) contains supplementary material, which is available to authorized users.

## Background

Insecticide-treated bed nets (ITNs) have been shown to effectively reduce malaria morbidity and mortality [1] and consequently, the last years have seen many intervention programs being put in place to distribute and promote the use of long-lasting insecticidal nets (LNs) in malaria endemic countries [2–5]. As a key strategy of the Roll Back Malaria initiative [6], LNs have been, and continue to be implemented as part of national malaria control programs around the world. With the increasing demand for treated bed nets and the ever present threat of the development of resistance to insecticides comes the need to ensure the best products are available for use. As a result, new bed net products are evaluated for efficacy, durability and operational acceptability prior to approval.

A very important feature of the efficacy testing of mosquito nets is the World Health Organization (WHO) cone bioassay [7]. It serves as a pivotal tool on which a lot of decisions made in efficacy studies are based. WHO instructs that nets would first have to meet the criteria of WHO cone bioassay before they are passed to go through phase II testing [7]. It is the recommended assay for testing the efficacy and irritant or excito-repellent properties of insecticide-treated substrates. It also plays a pivotal role in IRS as it is used to test formulations of insecticides on various substrates such as mud, cement, plywood and other materials commonly used for building [8]. In 2013, the WHO Pesticide Evaluation Scheme (WHOPES) published the latest version of the Guidelines for laboratory and field-testing of long-lasting insecticidal nets [7] which replaced the earlier version published in 2005 [9]. The document outlines the procedure for testing LNs and provides the current set of instructions for performing the WHO cone bioassay. Even though its purpose is to provide specific, standardized procedures for testing LNs to harmonize testing procedures in order to generate data for registration and labelling of such products [7], it leaves some important details in the instructions to the discretion of the personnel conducting the experiment, which could potentially influence the outcome of the test. One such detail is the angle at which the set-up should be held during exposure. To test samples of treated bed nets, the assay is set up by attaching WHO polyvinyl chloride (PVC) cones to the net sample and the two are usually secured together by two plastic panels with circular holes cut in them to accommodate the cone and expose the netting. These panels are held together by two metallic binder clips. The guidelines do not provide any information on the positioning of the assembly. The only mention of the angle is in the caption of the figure illustrating the cone assay, where it states that the holding board on which the assembly rests is slanted at an angle of 45°. The angle is completely ignored in the case of using the assay on sprayed surfaces where the PVC cone is taped to the surface of interest, in which case the angle could range from a flat table (0°), through an upright wall (90°) to an upside down ceiling (180°). The assay heavily depends on the mosquitoes making contact with the net or surface being tested. Due to the behaviour of mosquitoes, the angle of testing could lead to less contact with the surface, subsequently leading to significant fluctuations in the outcome of the assay.

On this background, the possible effects the angle of testing has on the outcome of the WHO cone bioassay on mosquito nets were investigated by performing the test following the recommended guidelines [7] at four different angles. The difference in behaviour as a function of angle was also evaluated.

## Methods

### Mosquitoes

Mosquitoes of two laboratory-bred *Anopheles* colonies were used in the experiment; the pyrethroid susceptible *Anopheles gambiae* Kisumu-1 and the pyrethroid resistant *Anopheles stephensi* STI. The Kisumu-1 (MRA-762) strain was obtained from the Malaria Research and Reference Reagent Resource Center in 2011. This is a standard strain used in cone bioassays to evaluate if a LN meets WHOPES specifications [8]. In addition, as occasionally new products are also evaluated against insecticide resistant mosquitoes, the pyrethroid resistant STI colony was also included. The colony was originally obtained from the London School of Hygiene and Tropical Medicine in 1971. The larvae were fed with finely ground fish food Tetramin (Tetra GmbH, Germany) and the resulting adults were maintained on 10 % sugar solution at temperature and relative humidity ranges of 26–28 °C and 60–74 %, respectively, in a 12:12 h day:night regime.

### Insecticide-treated nets

To account for the repellent/irritant effect, Olyset Plus^®^ and Netprotect^®^, impregnated with permethrin and deltamethrin, respectively were used. Olyset Plus is produced by Sumitomo Chemicals Co. Ltd. (Japan) and is made of knitted polyethylene thread incorporated with 2 % (w/w) permethrin combined with 1 % piperonyl butoxide. Netprotect, manufactured by BestNet A/S (Denmark), is also a polyethylene LN and it is impregnated with 0.18 % (w/w) deltamethrin.

### WHO cone bioassay

**Fig. 1:**
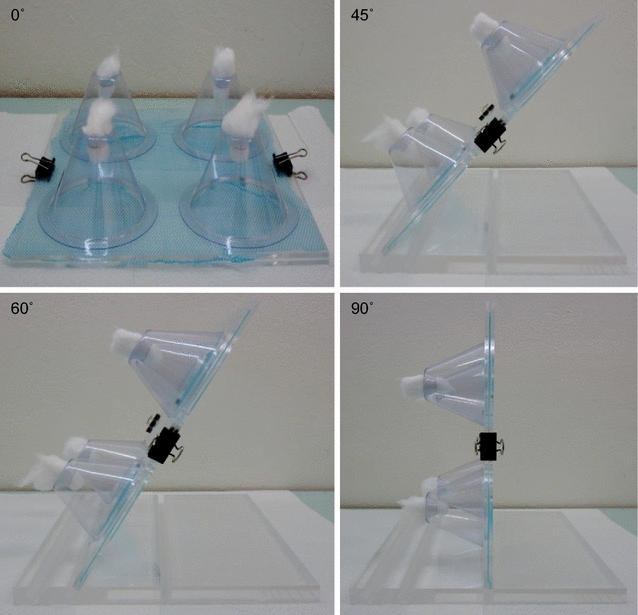
Cone bioassay of LNs. The holding board was slanted at different angles of 0°, 45°, 60° and 90°

The bioassay was performed according the WHOPES guidelines [7]. The set-up was prepared by cutting approximately 25 cm × 25 cm pieces of netting from each net type. Four WHO plastic cones were attached to each piece of net and held together by two plastic boards which were clamped together with two binder clips. The assembly was held at one of the test angles; 0° (flat on the table), 45°, 60° and 90° (Fig. 1). Using an aspirator, five to eight non blood-fed females aged two to five days were introduced into each cone and the holes were plugged by pieces of cotton. The mosquitoes were exposed for 3 min and subsequently transferred into labelled 150 ml plastic holding cups and provided with 10 % sugar solution. Knockdown and mortality were recorded 60 min and 24 h after exposure, respectively. Mosquitoes were scored as alive if they were able to fly, irrespective of the number of legs still intact and dead, or knocked down, if immobile or incapable of flying or standing in a coordinated manner [10]. An untreated net was used as a control and was tested each day the bioassay was performed. The bioassays were performed at temperature and relative humidity ranges of 25.9–29 °C and 58–73 %, respectively.

### Washing procedure

After the first round of testing, the net sample pieces were washed a total of 20 times each, following the WHO recommended procedure for washing nets for laboratory testing [7]. Individual net pieces were introduced into 0.5 l of deionized water in a 1 l beaker. Each net was washed in a separate beaker designated to that net to avoid cross-contamination. Just before washing, 1 g of the WHO-recommended soap “Savon de Marseille” was added and fully dissolved. The beaker was immediately put in an incubator set at 30 °C and shaken at 155 movements per minute for 10 min. The nets were then removed, rinsed twice in clean deionised water for 10 min at the conditions given above. After washing, they were dried at room temperature and stored at 27–29 °C in the dark between washes. Washing was done at a minimum of seven-day intervals to allow regeneration of insecticides. Testing was repeated as described above with the washed nets.

### Time spent in contact with the net

Based on the results from the angle experiments, mosquitoes were exposed to nets elevated at 60° or flat at 0° and their behaviour inside the cone was observed to evaluate the correlation between time spent in contact with the nets and mortality. Mosquitoes were introduced individually into the cone setup and observed for their behaviour during the 3 min of exposure. Within this period, recordings were made on the amount of time the mosquito spent flying around, resting on the net, on the cotton plug and on the side of the PVC cone. As in the experiment described above, mosquitoes from each strain were exposed to washed and unwashed pieces of Olyset Plus and Netprotect. A total of 15 individuals were tested for each combination of net product, net state (i.e. washed or unwashed), mosquito strain, and angle. To minimize the effect of the act of blowing the mosquitoes into the cone on the behaviour, each mosquito was gently blown onto the net.

### Data analysis

The data were analysed by generalized linear mixed-effects models (GLMM) in the freely available statistical software package R [11], version 3.1.2 and the R package lme4 [12, 13]. The day of testing was included as a random intercept in the models to account for correlations within the same day. The level of significance was set at *α* = 0.05.

Logistic regressions were used to assess the effect of the angle on knockdown and 24 h mortality. Angle 0° was used as the reference for comparison. In addition to the angle, strain and the net product were also included as fixed factors in the models and the data was analysed separately for washed and unwashed nets. The amount of time the mosquitoes spent resting on the net was analysed using a linear regression. Time spent was predicted by angle, mosquito strain, net product and state of the net (i.e. washed or unwashed). The R package lmerTest [14] was used to generate the p values for the estimates in the GLMMs.

## Results

### Effect of angle on knockdown and mortality

**Fig. 2:**
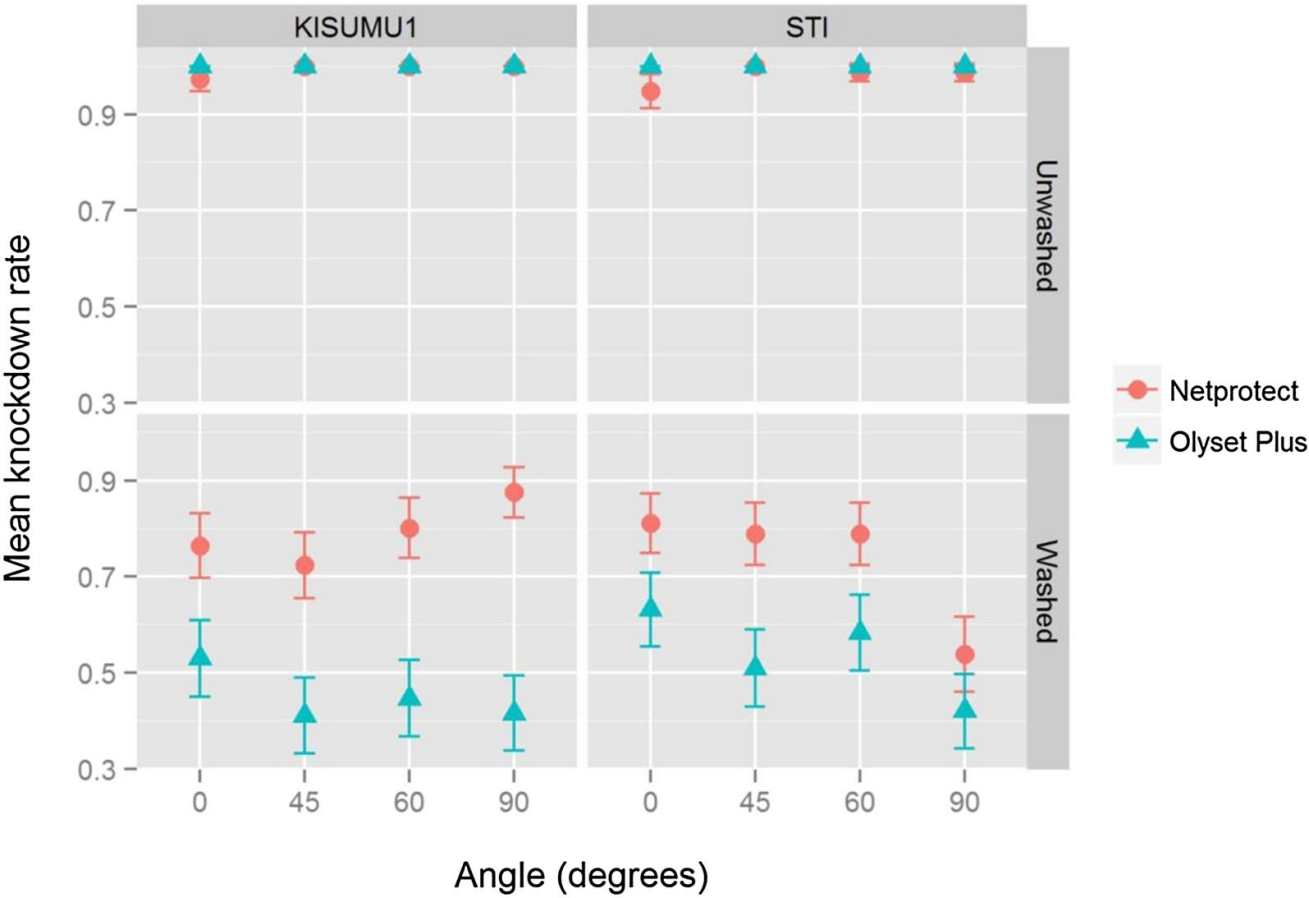
Knockdown rates at the various angles recorded in the KISUMU-1 and STI strains against washed and unwashed nets. The *points* and the *whiskers* represent mean knockdown rates and 95 % confident intervals, respectively

A minimum of 150 mosquitoes were exposed for each strain, net type, net state and angle combination. Table 1 summarizes the mortality recorded in the various groups. In the unwashed nets, knockdown rates were very high (Table 1; Fig. 2) and there was no difference detected between the two strains (odds ratio = 0.3, 95 % CI 0.01–1.03, p = 0.06). As shown in Table 2, in the washed nets, knockdown rates were significantly lower in Olyset Plus than in Netprotect (OR 0.3, 95 % CI 0.25–0.36, p < 0.001). Holding the test unit at 45° (OR 0.7, 95 % CI 0.5–0.9, p = 0.004) and 90° (OR 0.6, 95 % CI 0.4–0.7, p < 0.001) produced significantly lower knockdown rates as compared to 0°, but there was no statistically significant difference at 60° (OR 0.9, 95 % CI 0.7–1.1, p = 0.26). Figure 2 shows a plot of the mean knockdown rates recorded for each combination.

**Table 1:** Summary of the number of mosquitoes tested, knockdown and mortality recorded in the test groups

Strain	Net product	Net state	Angle (°)	No. tested	Knockdown and 95 % CI (%)	Mortality and 95 % CI (%)
KISUMU-1	Olyset Plus	Unwashed	0	153	100 (97.6–100)	90.2 (84.5–94.0)
			45	154	100 (97.6–100)	96.8 (92.6–98.6)
			60	154	100 (97.6–100)	98.1 (94.4–99.3)
			90	161	100 (97.7–100)	100 (97.7–100)
	Netprotect		0	151	97.4 (93.4–99.0)	86.8 (80.4–91.3)
			45	157	100 (97.6–100)	96.8 (92.8–98.6)
			60	155	100 (97.6–100)	98.1 (94.5–99.3)
			90	156	100 (97.6–100)	100 (97.6–100)
	Olyset Plus	Washed	0	152	53.0 (45.0–60.8)	13.3 (8.7–19.6)
			45	151	41.1 (33.6–49.0)	14.6 (9.8–21.1)
			60	150	44.7 (36.9–52.7)	8.0 (4.6–13.5)
			90	154	41.6 (34.1–49.5)	5.2 (2.7–9.9)
	Netprotect		0	153	76.5 (69.2–82.5)	26.8 (20.4–34.3)
			45	163	72.4 (65.1–78.7)	54.6 (46.9–62.1)
			60	155	80.2 (73.2–85.6)	44.8 (37.3–52.7)
			90	153	87.6 (81.4–91.9)	41.8 (34.3–49.8)
STI	Olyset Plus	Unwashed	0	155	100 (97.6–100)	70.3 (62.7–77.0)
			45	166	100 (97.7–100)	86.1 (80.1–90.6)
			60	155	100 (97.6–100)	87.1 (80.9–91.5)
			90	161	100 (97.7–100)	77.0 (69.9–82.8)
	Netprotect		0	154	94.8 (90.1–97.3)	42.2 (34.7–50.1)
			45	165	100 (97.7–100)	77.0 (70.0–82.7)
			60	153	98.7 (95.4–99.6)	73.9 (66.4–80.2)
			90	154	98.7 (95.4–99.6)	61.7 (53.8–6.90)
	Olyset Plus	Washed	0	152	63.2 (55.3–70.4)	4.0 (1.8–8.3)
			45	151	51.0 (43.1–58.8)	2.0 (0.7–5.7)
			60	151	58.3 (50.3–65.8)	2.7 (1.0–6.6)
			90	157	42.0 (34.6–49.9)	2.6 (1.0–6.4)
	Netprotect		0	153	81.2 (74.3–86.6)	8.4 (5.0–13.9)
			45	152	78.9 (71.8–84.7)	12.5 (8.15–18.7)
			60	152	78.9 (71.8–84.7)	13.8 (9.2–20.2)
			90	158	53.8 (46.0–61.4)	6.3 (3.5–11.3)

The total number of mosquitoes tested is the result of at least 27 replicates for each treatment

**Table 2:** A summary of the outputs from the logistic regression models explaining the predictors of mortality and knockdown

Net state	Outcome	Explanatory variable	Odds ratio	95 % CI	*p* value
Unwashed	Mortality	Product (Olyset Plus)	1.9	1.5–2.5	<0.001
		Strain (STI)	0.1	0.04–0.2	<0.001
		Angle (45°)	3.3	1.7–6.3	0.001
		Angle (60°)	3.1	1.7–5.9	<0.001
		Angle (90°)	6.0	1.9–18.5	0.002
Washed		product (Olyset Plus)	0.5	0.3–0.8	0.01
		Strain (STI)	0.06	0.03–0.12	<0.001
		Angle (45°)	2.7	1.7–4.3	<0.001
		Angle (60°)	1.9	1.1–3.1	0.01
		Angle (90°)	1.4	0.8–2.3	0.212
Washed	Knockdown	Product (Olyset Plus)	0.3	0.25–0.36	<0.001
		Strain (STI)	1.1	0.89–1.3	0.5
		Angle (45°)	0.7	0.5–0.9	0.004
		Angle (60°)	0.9	0.7–1.1	0.26
		Angle (90°)	0.6	0.4–0.7	<0.001

Netprotect, KISUMU-1 and the angle 0° were the reference levels (intercepts) in the coefficients. No model was ran on knockdown in the unwashed nets due to the high levels of knockdown observed

**Fig. 3:**
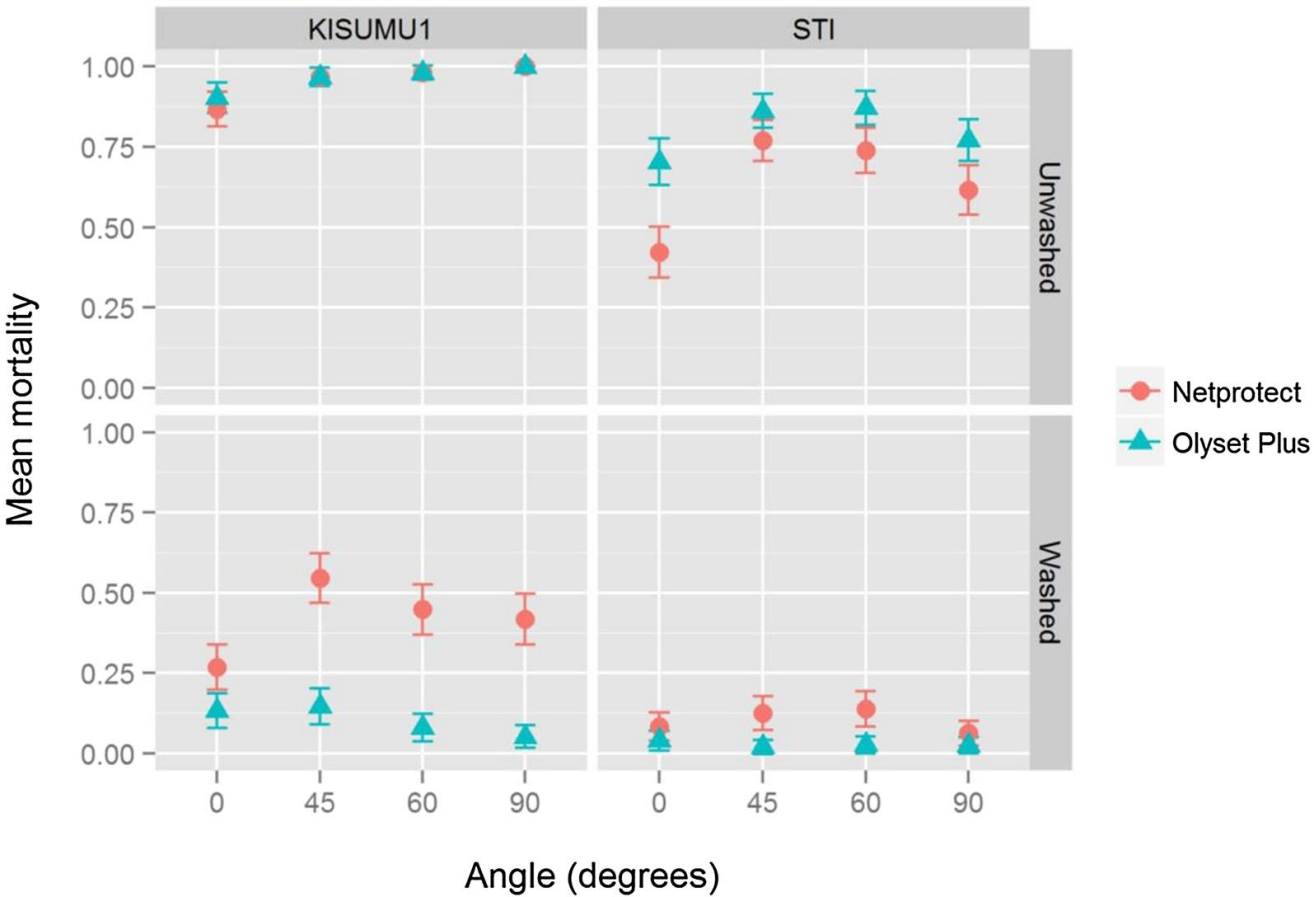
Mortality at the various angles recorded in the KISUMU-1 and STI strains against washed and unwashed nets. The *points* and the *whiskers* represent mean mortalities and 95 % confident intervals, respectively

Mortality was generally lowest at 0° (Fig. 3; Table 1). The highest mortality was usually at 45° or 60°, and a closer look showed that statistically, there was no significant difference in the mortality between the two angles in washed (OR 0.9, 95 % confidence interval = 0.5–1.5, p = 0.6) and unwashed nets (OR 1.0, 95 % CI 0.6–1.5, p = 0.9). On the unwashed nets, mosquitoes were more likely to die on Olyset Plus (OR 1.9, 95 % CI 1.5–2.5, p < 0.001) than on Netprotect (Table 2), while this observation was reversed in the washed nets (OR 0.5, 95 % CI 0.3–0.8, p = 0.008). Mortality was significantly increased when the tests were performed at 45° (OR 3.3, 95 % CI 1.7–6.3, p = 0.001), 60° (OR 3.1, 95 % CI 1.7–5.9, p < 0.001) and 90° (OR 6.0, 95 % CI 1.9–18.5, p = 0.002) as compared to 0°. There were no observed interactions between any of strain, angle and net product. After 20 washes there was no statistically significant difference between the mortality obtained at 0° and 90° (OR 1.4, 95 % CI 0.8–2.3, p = 0.212). However, mortality was significantly higher at 45° (OR 2.7, 95 % CI 1.7–4.3, p < 0.001) and 60° (OR 1.9, 95 % CI 1.1–3.1, p = 0.01). There was a significant interaction between the net product and the angle of assay (interaction term OR at 45° = 0.3, p < 0.001; 60° = 0.2, p < 0.001; 90° = 0.2, p < 0.001; Likelihood-ratio test: χ^2^ = 14.4, p = 0.002), indicating that the effect of angle on mortality is different for the different net products.

The ambient temperature and relative humidity measured during testing did not have any significant effect on the test results in neither the washed (temperature: OR 1.0, 95 % CI 0.4–2.8, p = 1.0; relative humidity: OR 0.8, 95 % CI 0.6–1.0, p = 0.10) nor the unwashed (temperature: OR 0.9, 95 % CI 0.5–1.9, p = 0.86; relative humidity: OR 1.0, 95 % CI 0.8–1.2, p = 0.91) nets.

### Time spent in contact with the net

**Fig. 4:**
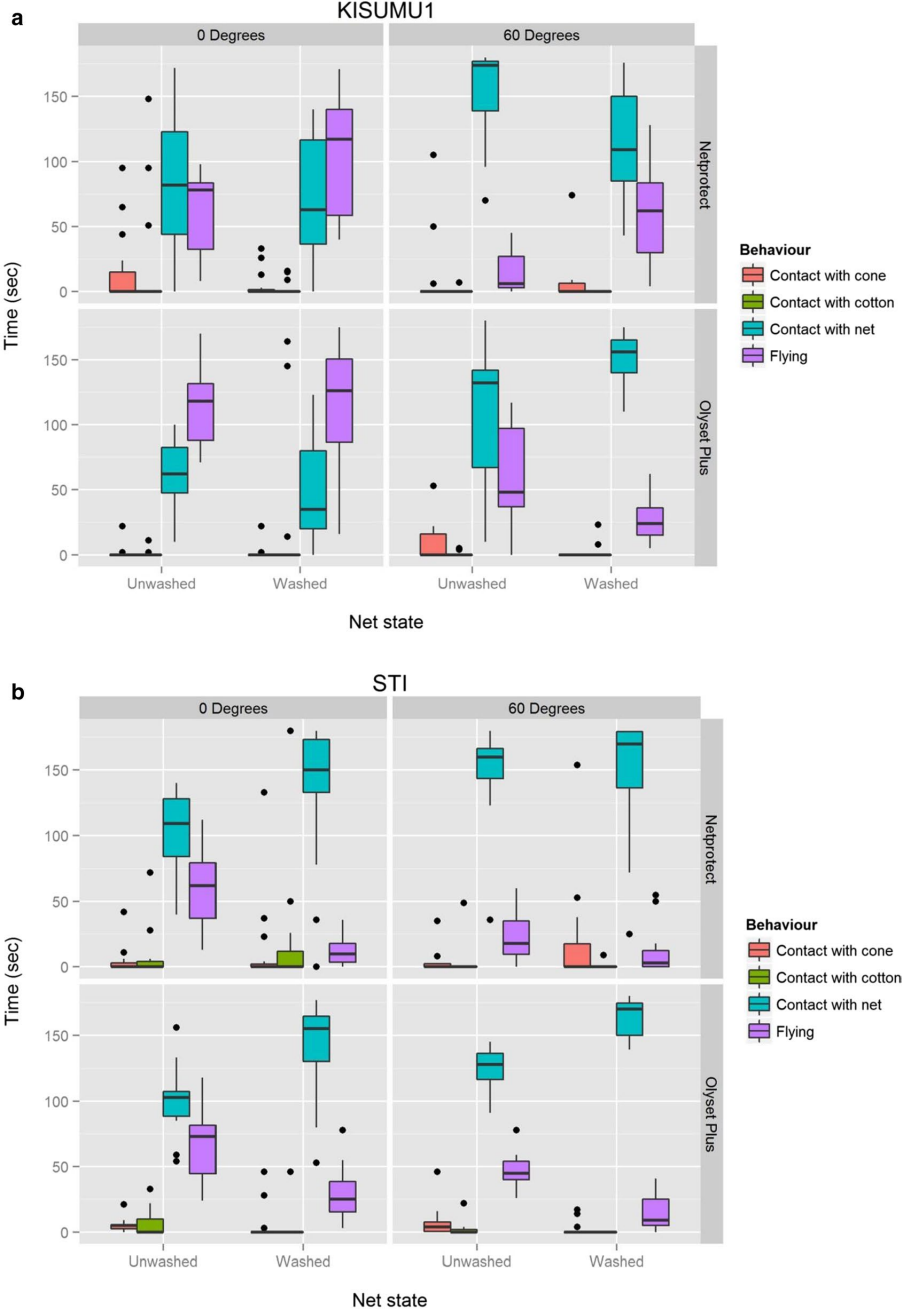
Boxplot of the time distribution in the observational experiment in KISUMU-1 (**a**) and STI (**b**). The *boxes* represent the interquartile distances (IQD), while the *center lines* through *each box* show the medians. The *dots* indicate outliers and the *whiskers* extend to the extreme values of the data, calculated as ±1.5 × IQD from the median

**Fig. 5:**
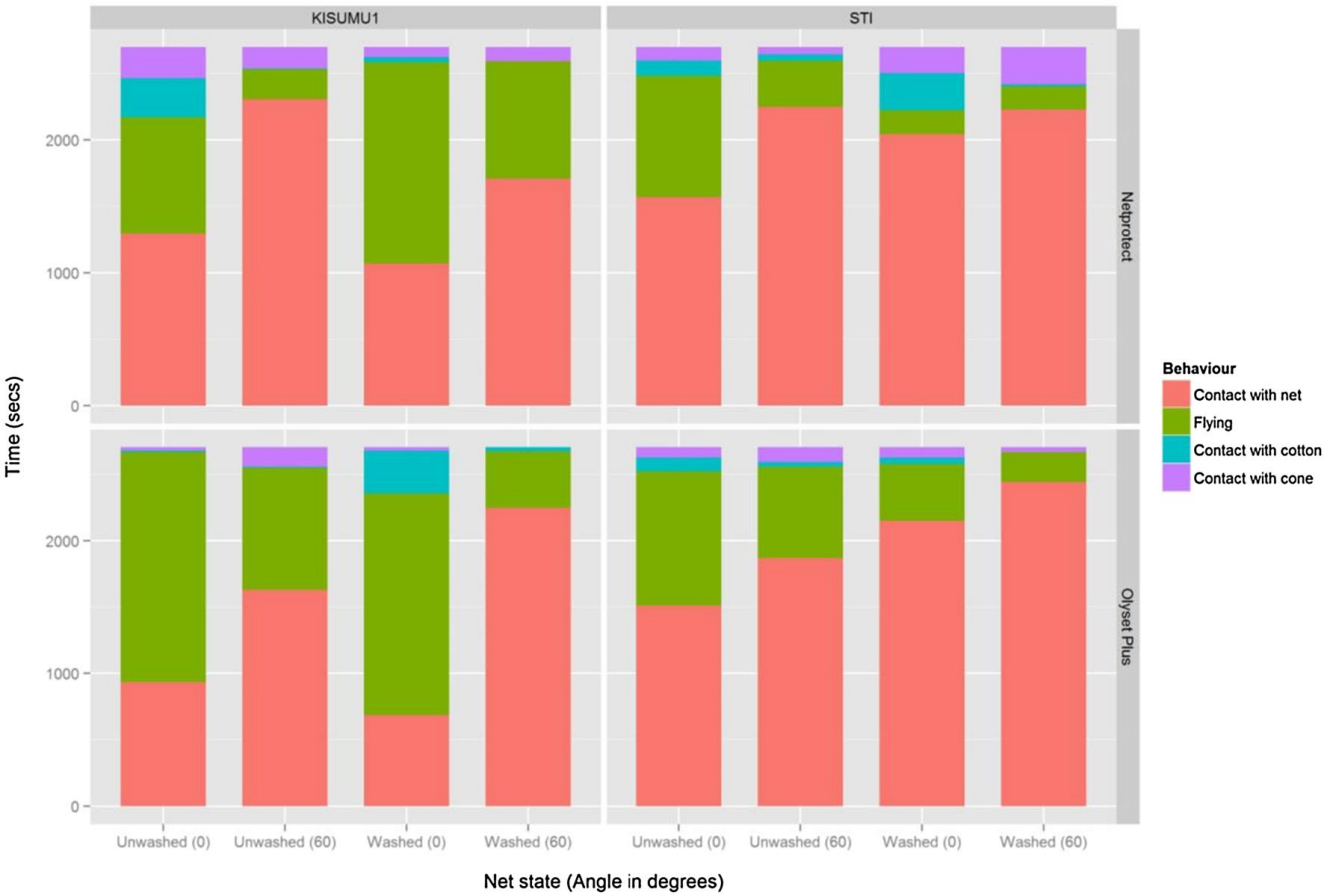
Proportion of the cumulative time the mosquitoes spent flying, resting on the net, cone and cotton within the 3 min of exposure

This experiment was carried out in order to examine whether the lower mortality rates observed at 0° could be explained by the amount of time the mosquitoes spent resting on the net samples. The two angles that produced the highest and the lowest mortality values were chosen. The highest mortalities were recorded at 45° and 60°, but because there was no statistically significant difference between the two angles, 60° was chosen, while the lowest mortality was recorded at 0°. A total of 240 mosquitoes were tested; 15 individuals from each strain against washed and unwashed pieces of the two net products at 0° and 60°. Cumulatively, mosquitoes spent more time in contact with the net at 60° (16,682 s) than at 0° (11,259 s) and more time in flight at 0° (8321 s) than at 60° (3883 s). They also spent more time on the cotton at 0° (1212 s) than at 60° (148 s). Figures 4 and 5 show graphical presentations of how both strains spent the time inside the cones. Indeed, the multiple linear regression model (Table 3) showed that mosquitoes spent more time in contact with the net at 60° than at 0° (coefficient = 44.8, 95 % CI 34.3–55.3, p < 0.001). The effect of the mosquito strain was also significant, with the resistant STI strain spending more time on the net than the susceptible Kisumu-1 (coeff = 24.9, 95 % CI 2.8–47.1, p = 0.05). The type of net (coeff = −11.3, 95 % CI −31.7 to 9.0, p = 0.3) and whether washed or not (coeff = 12.2, 95 % CI −2.4 to 26.7, p = 0.1) did not have a significant influence. A 3-min side-by-side video showing mosquitoes exposed at 0° and 60° is provided as an Additional file 1.

**Table 3:** The output of the linear regression model predicting the time spent by the mosquitoes resting on the net

Explanatory variable	Coefficient	p value	95 % CI
Product	−8.4	0.1	−19.0 to 2.2
Strain	34.9	<0.001	24.3 to 45.4
Angle	0.8	<0.001	0.6 to 0.9
Net state	−10.1	0.06	−20.7 to 0.5

Netprotect, KISUMU1 and the angle 0° were the reference levels in the coefficients

## Discussion

The results from the current study provide evidence that the angle at which the WHO cone bioassay is performed considerably affects the time mosquitoes spend in contact with the net, and subsequently 24 h mortality. The cone assay is heavily depended on as the main test for the determination of the bioefficacy in terms of insecticidal activity and irritant or excito-repellent properties throughout the three WHOPES phases of the efficacy assessment of LNs. It serves as the first test in phase I trials and is used to generate information on the efficacy and wash-resistance of the nets and to assess the interactions between the insecticide and the netting fiber such as regeneration time [7]. An assay of such importance requires specific instructions on details pertaining to the procedure rather than leaving it to the performer’s convenience. The importance of the angle at which the assay is performed has been largely ignored, although this should be considered an essential feature of the assay. According to WHO [15], the cone assay is recommended as the assay of choice for insecticidal activity because it directly measures the amount of insecticide available to contact and kill mosquitoes and a net is expected to produce a mortality of ≥80 or ≥95 % knockdown to fulfil the WHO efficacy requirements. This implies that the time the mosquitoes spend in contact with the net is very important for this purpose. Yet, as data from this study show, for the assay to effectively measure this parameter, the guidelines need to be updated to incorporate the angle of testing. Due to the assay’s function of measuring irritancy, it makes sense that the WHO cone has enough room to accommodate the behaviour of irritated mosquitoes. On the other hand, this could also provide enough space for flying which could affect residual efficacy measurements. In the observational experiment, no mosquitoes were observed to have spent all the 3 min in flight but six individuals were recorded, all at 0°, that did not spend any time resting on the net and 11 that rested for less than 30 s. Given that these mosquitoes were introduced individually, factoring in the disturbance due to the presence of other mosquitoes as is the case in the standard procedure and the presence of excito-repellent effects could possibly result in more mosquitoes making no contact at all with the test material when testing on a flat surface. Currently, a net which fails to meet the requirements of the cone assay in phase I undergoes the tunnel test to measure the mortality and success of blood-feeding of host-seeking mosquitoes. This is a step to ensure that the efficacy of nets are not underestimated due to high excito-repellent effects of certain insecticides [7]. The tunnel test is very instrumental for this purpose due to its ability to capture the activity of slow acting compounds [16]. A major downside of the tunnel test however, is the use of live animals as baits which may vary in how attractive they are to mosquitoes. The baits are kept in very small confinements in such a way that they cannot move or feed within the 12–15 h exposure period. Although there are guidelines in place to ensure that animals used for testing are not mistreated, they could be subjected to unpleasant and cruel violations if the test is not performed properly. While the two assays play very important roles in the evaluation process, optimizing and adjusting the methodology of the cone assay to improve reliability would save energy and resources and ensure the tunnel test is only turned to when actually needed. If cone tests are used for intrinsic insecticidal activity when screening, for example, for new compounds or formulations, separate assays could also be developed such that the setup of the assay reduces the space inside the cone to minimize flying and force contact with the test material.

**Fig. 6:**
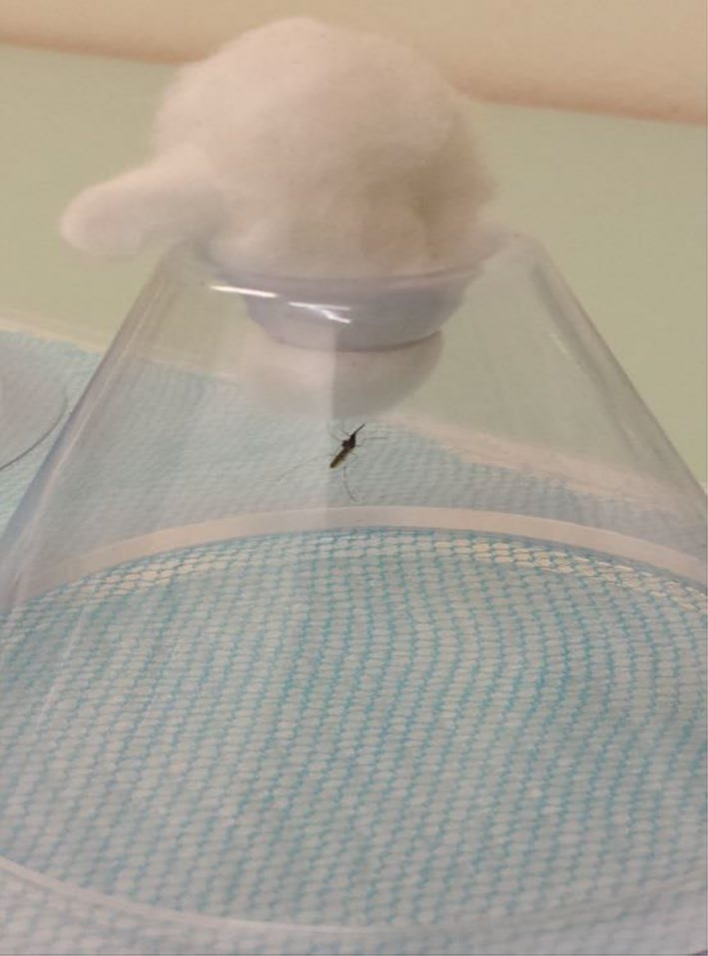
Some mosquitoes spent a substantial amount of time resting on the cotton and not in contact with the net

In addition to mosquitoes flying around, individuals that spend time on the cone and the cotton plug were also recorded in both experiments (Fig. 6), therefore avoiding contact with the net for significant periods of time. This is one area which could also be improved, design-wise, to minimize the non-treated surfaces inside the cone. In an attempt to reduce the chances of mosquitoes resting on the cone instead of the treated nets, some studies [17] put net flaps inside the cones. While this could work well and ensure increased contact with the net, it reduces the comparability of results between studies and laboratories. The wire-ball test, an alternative to the cone assay, overcomes the problem of mosquitoes resting on untreated surfaces. It consists of a cubical 15 cm × 15 cm × 15 cm or two intersecting circles of about 15 cm diameter wire frame around which the piece of netting is wrapped [8], thereby reducing the area of untreated surfaces inside the exposure space. However, before this test can be used, it has to be calibrated with the WHO cone assay [8].

Aside from the evaluation of LNs, the cone assay is also used for testing the insecticide bioavailability and residual activity of insecticide-treated substrates such as housing materials after indoor residual spraying (IRS) [8] and durable wall linings [18] in small-scale (Phase II) and large-scale (Phase III) WHOPES field trials. For this purpose, the cones are attached to the walls and ceilings of the experimental huts or houses. This automatically results in different angles that could range from 0° (flat) to 180° (upside down). Although in this study only nets were tested and the effects at 180° were not evaluated, it is expected that the variation in the angles will result in a similar outcome in other substrates. The mosquitoes are exposed for a longer period of 30 min, but an increased number of 10 mosquitoes could result in individuals being affected by both the angle and the presence of other flying mosquitoes, thereby making less contact with the treated surface. This situation could be even worse in the presence of an irritant. The observation that the susceptible Kisumu-1 strain spent less time in contact with the net as compared to the resistant STI strain (Fig. 5) suggests that any irritancy property of the insecticide had a stronger effect on the susceptible mosquitoes.

While the data from the current study show that mosquitoes tend to spend more time on the net at 60°, there is a valid argument for performing the assay at 0°. A recent study showed that most of the activities and net contact of host-seeking mosquitoes occur on the top side of the net [19]. Therefore, testing at 0° could provide a realistic assessment of the kill ability of the nets.

Apart from the angle, another detail which could influence the amount of time the mosquito spends in contact with the net is how they are blown into the cone. It is not yet defined whether the mosquitoes have to be blown directly onto the net or rather blown to fly freely in the cone. This could affect the resting or flying behaviour of the mosquitoes in the cone and could influence the amount of time they spend in touch with the net. Making initial contact with the net upon introduction into the cone could also result in mosquitoes picking up insecticide, which could make a difference in the observed mortality, especially in the case of testing high concentrations of insecticide. This hypothesis would be in agreement with Sternberg et al. [20] who also suggested that accidental contact of mosquitoes to treated materials beyond exposure periods could alter the outcome of the assay.

## Conclusion

From the results of this study, the inclination at which the test is performed is rather an important component of the assay and changing the angle leads to inconsistencies in the outcome. The WHOPES guidelines should be explicit in defining a working angle in all instances where the cone assay is used for the evaluation of treated substrates. From the data shown here, performing the test at an inclined angle results in more contact with the treated surface than on a flat surface and the 45° implicitly suggested in the WHOPES guidelines is set at a comfortable working slope. Clearly stating this angle in the WHOPES guidelines as the standard working angle is highly recommended.

## Additional file

10.1186/s12936-016-1303-9 Side-by-side video showing how two mosquitoes exposed at 60° (left) and 0° (right) behaved in the 3 min of exposure.
